# Epistatic Interactions Alter Dynamics of Multilocus Gene-for-Gene Coevolution

**DOI:** 10.1371/journal.pone.0001156

**Published:** 2007-11-07

**Authors:** Andy Fenton, Michael A. Brockhurst

**Affiliations:** School of Biological Sciences, University of Liverpool, Liverpool, United Kingdom; The Wellcome Trust Sanger Institute, United Kingdom

## Abstract

Fitness costs associated with resistance or virulence genes are thought to play a key role in determining the dynamics of gene-for-gene (GFG) host-parasite coevolution. However, the nature of interactions between fitness effects of multiple resistance or virulence genes (epistasis) has received less attention. To examine effects of the functional form of epistasis on the dynamics of GFG host-parasite coevolution we modified a classic multilocus GFG model framework. We show that the type of epistasis between virulence genes largely determines coevolutionary dynamics, and that coevolutionary fluctuations are more likely with acceleratingly costly (negative) than with linear or deceleratingly costly (positive) epistasis. Our results demonstrate that the specific forms of interaction between multiple resistance or virulence genes are a crucial determinant of host-parasite coevolutionary dynamics.

## Introduction

Parasites are ubiquitous in biological systems, and the coevolutionary arms races between hosts and their parasites are thought to drive a wide range of ecological and evolutionary phenomena [1]. One of the primary frameworks developed to study host-parasite coevolutionary dynamics is the gene-for-gene (GFG) model, which emerged from empirical studies on plant-pathogen dynamics [2], [3]. Under this model, the outcome of contact between a host and a parasite (i.e., whether infection occurs or not) depends on the combination of their genotypes. In the simplest case both host and parasite are assumed to have a single locus with two possible alleles: the host has either a resistant or susceptible allele and the parasite has either a virulent or avirulent allele. The parasite is effectively ahead in the interaction, such that susceptible hosts may be infected by either parasite genotype and virulent parasites may infect both host genotypes; hence, three out of the four possible combinations of host and parasite genotypes result in successful infection. Under this scenario, the dynamic interactions between hosts and parasites result in an escalatory arms race, with increases in the frequencies of resistant and virulent alleles. However, if there are costs associated with harbouring resistant or virulent alleles then this can prevent escalation, resulting in either stable dynamics, or sustained coevolutionary cycles. This simple model has been extended to multilocus models [4], [5], where costs can prevent the occurrence of generalist parasites (‘super-races’) that infect all host genotypes, resulting in the occurrence of sustained, potentially chaotic fluctuations in host and parasite genotypes [5].

**Figure 1:**
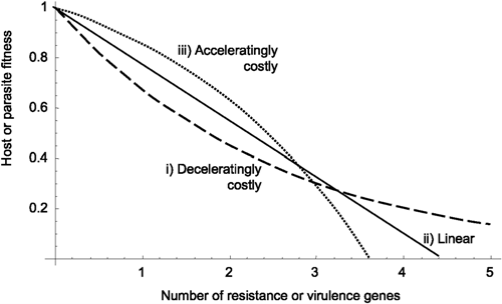
Host (and parasite) fitness curves showing three possible relationships between the number of resistance (or virulence) alleles and host (or parasite) fitness. The lines all have the same overall fitness when summed across all 5 genes.

A fundamental assumption of multilocus GFG models that has not previously been examined is the nature of the costs associated with host resistance or parasite virulence and how their fitness effects interact (i.e., the functional form of epistasis). Multilocus models typically assume either a multiplicative [4] or a negative exponential, deceleratingly costly [5] relationship between the number of resistance (or virulence) genes and host (or parasite) fitness (Fig 1). However, as yet there has been no mechanistic justification for either assumption and, since quantifying fitness effects of epistatic interactions between multiple resistance or virulence genes empirically is notoriously difficult, it is not known which types of epistasis tend to naturally occur in the wild [for related discussion see 6]. Nevertheless, alternative forms of epistasis are possible (Fig 1). Furthermore, there is no reason to assume that both host and parasite will have the same form of epistais (e.g., the host's curve might be deceleratingly costly whereas the parasite's may be acceleratingly costly). As has been shown in models of host-parasite evolution (rather than co-evolution, as considered here), the specific nature of the host's cost curve can have important consequences for the evolution of resistance [7], [8], [9]. Therefore in a coevolutionary model it is likely that the shapes of host-parasite epistasis curves will determine the evolutionary potential of antagonists and hence the dynamics of coevolution. Here we use a modified version of Sasaki's multilocus gene-for-gene model [5] to explore how different combinations of host and parasite epistasis alter the host-parasite coevolutionary dynamics.

## Methods

Our multilocus GFG model follows that of Sasaki [5]. However, we generalise the host and parasite fitness functions to incorporate alternative epistasis curves. Here we describe our modifications to the Sasaki model; full details of the general model structure can be found in the online appendix (Supplementary Methods S1). In the Sasaki model the fitness of host genotype *s* was given as:
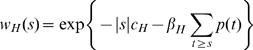
(1)where |*s*| is the number of resistance genes the host has, *c*_H_ is the cost incurred per resistance gene and *β*_H_ the selection intensity for a unit increase in mean parasite load (i.e., the cost to the host of being infected by a single parasite). The summation is the sum of parasite frequencies (of genotypes *t*) that can infect host genotype *s*. Equation 1 explicitly describes a negative exponential (decelerating) cost function between the number of resistance genes and the fitness of the host in the absence of the parasite. In other words, each successive resistance gene is less costly than the previous ones. The above fitness function may be generalised to:
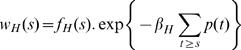
(2)where *f*_H_(*s*) is a general function describing the cost to the host of having |*s*| resistance genes. Here we assume three forms of this relationship:

1. Deceleratingly costly (Fig 1i): *f_H_* (*s*) = exp{−|*s*|*c_H_*}, as in Sasaki [5],
2. Linear (Fig 1ii): *f_H_* (*s*) = 1−|*s*|*k* and
3. Acceleratingly costly (Fig 1iii): *f_H_* (*s*) = 1+*q*(1−exp{|*s*|*c_H_*})

The parameters *k* and *q* are constants which were adjusted to ensure that all three curves had the same overall fitness value across all loci for a given value of *c*_H_, constrained so that fitness can never be negative. Therefore we can be sure that any differences in coevolutionary dynamics between the three curves arise purely as a result of the shape of the cost function, rather than as a result of one curve having a higher or lower mean fitness than another.

Finally, we assumed a similar general fitness function for a parasite of genotype *t*:
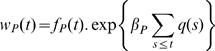
where *β*_P_ is the selection intensity for a unit increase in mean host availability and the summation is the sum of host frequencies (of genotypes *s*) that can be infected by parasite genotype *t*. As before, *f*_p_(*t*) is a general function describing the cost to the parasite of having |*t*| virulence genes, which takes forms similar to those describing the host's fitness functions above, with a given cost of *c*_P_ per virulence gene. Given this framework, both hosts and parasites can independently take one of three fitness curve shapes (deceleratingly, linearly or acceleratingly costly), leading to nine qualitatively different combinations of host and parasite fitness curves. We explored the consequences of these different combinations by running the multilocus GFG model at all combinations of both high and low host resistance and parasite virulence costs (*c*_H_ and *c*_P_); the results for all combinations of cost functions are given in the online Supplementary Material (Figs S1, S2, S3, S4, S5, S6), but here we just present the key results. In particular, host cost structure has little impact on coevolutionary dynamics and so here we just present the results of varying parasite cost structure.

## Results and Discussion

**Figure 2:**
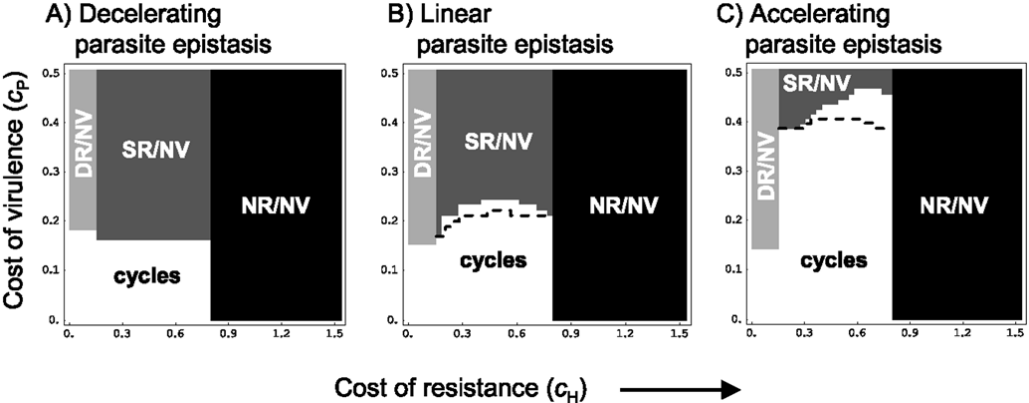
Phase diagram of coevolutionary dynamics, showing coevolutionary outcomes of different combinations of *c*_H_–*c*_P_. Here we follow the terminology of Sasaki (2000) and define regions to be of either coevolutionary cycles (“cycles”; no shading), double-resistance-no-virulence (“DR/NV”, where all hosts in the population have two resistance genes and no parasites have virulence genes; light shading), single-resistance-no-virulence (“SR/NV”, where all hosts have a single resistance gene and no parasites have virulence genes; intermediate shading) or no-resistance-no-virulence (“NR/NV”, where no hosts have resistance genes and no parasites have virulence genes; dark shading) occur. (A) the parasite has deceleratingly costly epistasis, (B) the parasite has linear epistasis and (C) the parasite has acceleratingly costly epistasis. In all cases the host has deceleratingly costly epistasis. The dashed lines show the boundary separating the regions where every simulation out of 20 replicates resulted in coevolutionary cycles (below the line) and where at least one of the replicate simulations resulted in a static single-resistance-no-virulence equilibrium (above the line).

**Figure 3:**
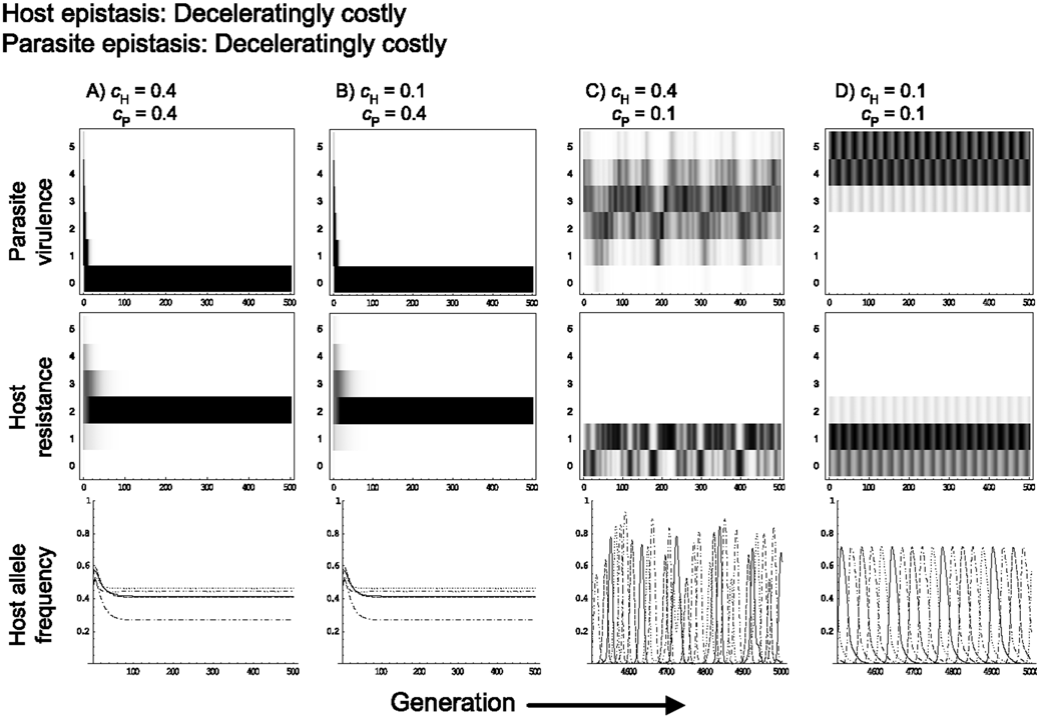
Coevolutionary host and parasite trajectories for different host (*c*_H_) and parasite (*c*_P_) costs, assuming deceleratingly costly epistasis for both the host and the parasite. The upper and middle panels show the frequency distributions over time of the number of virulence alleles in the parasite population and the number of resistance alleles in the host population, respectively. The bottom panels show the change in frequency of the host resistance alleles at each locus, where different line styles represent different loci. In all cases σ = 0.2, β_H_ = 1, β_P_ = 1 and μ, the mutation rate at each locus, was 2×10^−5^ per generation.

When both hosts and parasites are subject to deceleratingly costly epistasis, as shown by Sasaki [5], the magnitude of the resistance and virulence gene costs can greatly affect the host-parasite coevolutionary dynamics (Fig 2A). If the cost of virulence in the parasite is relatively high then static coevolutionary equilibria can occur with, for example, no virulence in the parasite population and single- (Fig 3A) or double- (Fig 3B) resistance polymorphism in the host. However, if the costs of virulence and resistance are relatively low then coevolutionary cycles can occur, where the number of resistance and virulence genes cycle endlessly (Figs 2A, 3C, 3D). These cycles are effectively repeated coevolutionary arms races, where host resistance and parasite virulence escalate, followed by crashes. This is most noticeable if both *c*_P_ and *c*_H_ are small, where regular coevolutionary fluctuations occur, with the host cycling through low levels of resistance and the parasite cycling through high levels of virulence (Fig 3D). However, higher values of *c*_H_ lead to more chaotic coevolutionary dynamics (Fig 3C), with the parasite cycling through the full possible range (0 to 5 virulence genes). At very high resistance costs the system reaches a stable state in which there is no virulence or resistance (Fig 2A).

**Figure 4 pone-0001156-g004:**
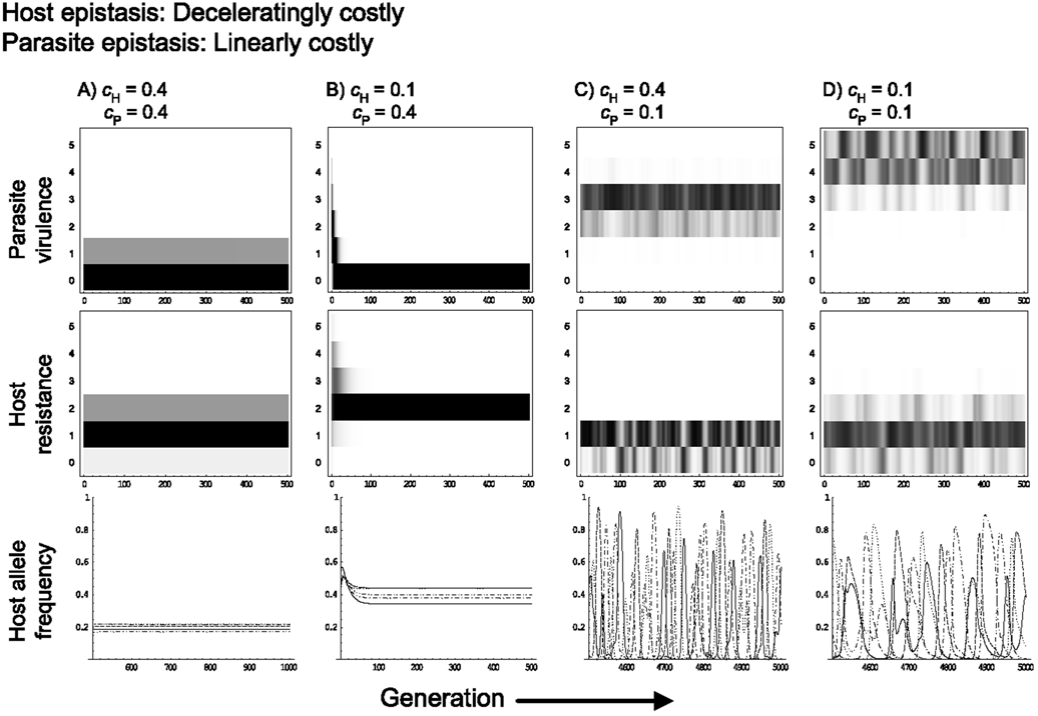
As for Figure 3, assuming deceleratingly costly epistasis for the host and linear epistasis for the parasite.

In general, altering the type of epistasis between host resistance genes has little impact on coevolutionary dynamics (see Figs S1, S2, S3, S4, S5, S6). However, altering the parasite's form of epistasis can affect both the quantitative and qualitative outcome of coevolution. Broadly speaking, the coevolutionary dynamics observed with linear epistasis for the parasite are quantitatively similar to those seen with deceleratingly costly epistasis (see Figs 2B, 4, S1, S2, S4 and S5), although linear epistasis tends to slightly increase the region under which coevolutionary cycles are observed (Fig 2B). Furthermore linear epistasis can change stable coevolutionary cycles into chaotic fluctuations for low host resistance and parasite virulence costs (Fig 4D).

**Figure 5:**
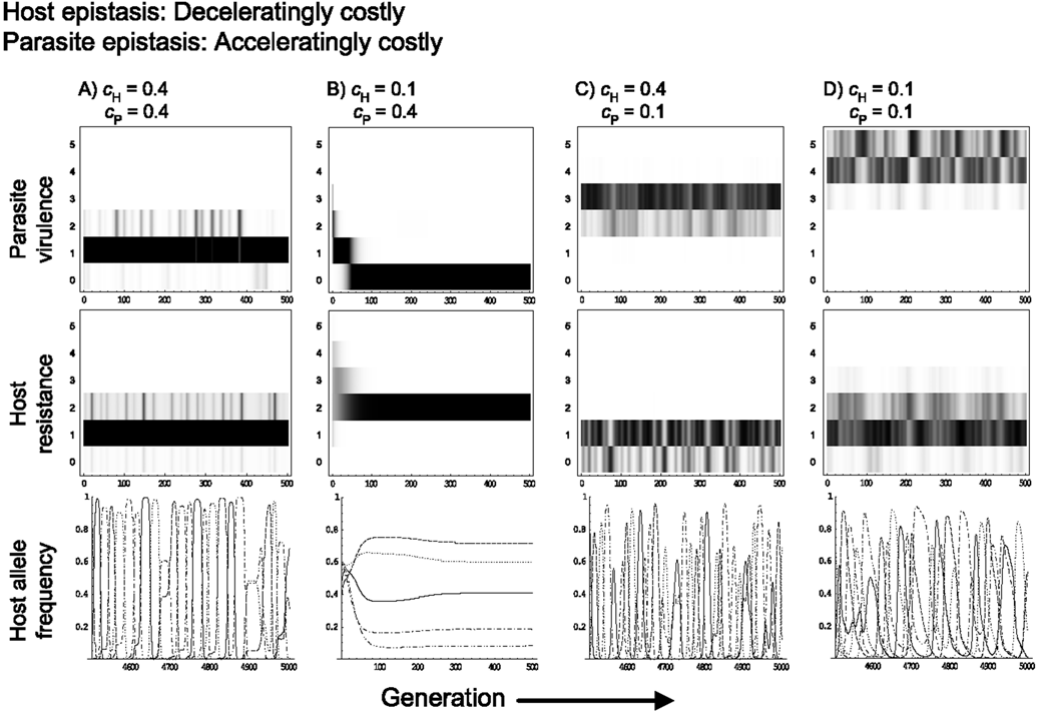
As for Figure 3, assuming deceleratingly costly epistasis for the host and acceleratingly costly epistasis for the parasite.

However, acceleratingly costly epistasis can greatly increase the parameter combinations under which coevolutionary cycles are observed; even if the cost to the parasite is relatively high, coevolutionary fluctuations can be maintained (Figs 2C, 5A, S3A, S6A) when they would otherwise not occur under conditions of linear or deceleratingly costly epistasis. In general the increased tendency for coevolutionary cycles under acceleratingly costly epistasis occurs at the expense of the region where single-resistance-no-virulence occurs (Fig 2C; Fig 5A). Hence, accelerating epistasis can maintain parasite virulence when it would otherwise not occur and the average virulence of the parasite population under acceleratingly costly epistasis tends to be higher than that observed under linear or deceleratingly costly epistasis (Fig 5A). Overall, although the type of the host epistasis does not greatly affect host-parasite coevolutionary dynamics, the type of the parasite epistasis is important, determining the presence and range of coevolutionary cycles, and whether the cycles are regular or chaotic. The primacy of parasite epistasis in our model is likely to be in part due to the nature of the GFG interaction, which assumes that parasites have the upper hand.

A further interesting point to note is that under acceleratingly costly epistasis stochastic factors become important in determining coevolutionary dynamics. Although sustained coevolutionary fluctuations can occur at high virulence costs (*c*_P_), it is also possible for static, single resistance equilibria to occur, and this tendency increases as *c*_P_ increases (Fig 2C). This is because at high virulence costs it is possible for all virulence alleles to fade out due to stochastic effects (in our model these arise from the random allocation of initial genotype frequencies at the start of each model run), thereby preventing the occurrence of sustained coevolutionary cycles. However, if virulence alleles survive in the parasite population this maintains the arms race and coevolutionary cycles occur. The impact of such stochastic effects appears less crucial to coevolutionary dynamics under deceleratingly, or even linearly, costly epistasis.

Our results suggest that the type of epistasis, along with the magnitude of the costs (i.e., c*_H_* and c*_P_*), are crucial determinants of evolutionary potential. Evolutionary potential appears to increase with the transition from decelerating to accelerating epistasis. This alters the balance of the coevolutionary arms race as evidenced, first, by the broader range of cost values over which dynamical coevolution is observed with accelerating epistasis, and second by examining the effects of mismatched host and parasite epistasis types on coevolutionary dynamics. For instance, when host epistasis is decelerating there is a transition from stable to chaotic cycles under low *c*_P _and *c*_H_ as parasite epistasis is changed from decelerating to linear or accelerating (Figures 3–5). Accelerating epistasis acts to buffer the fitness effects of resistance or virulence genes, which appears to confer a significant evolutionary advantage and can even compensate for high costs.

While costs of certain individual resistance genes have been quantified [see for example 10], quantifying epistasis between multiple genes is far more difficult, and there is little information informing us as to what shapes to expect in natural host-parasite systems [6]. However if, as has been recently suggested, there is a negative correlation between the average sign of epistasis and genomic complexity [11], then it may be possible to predict the type of epistasis based upon genome size. Small genomes tend to display positive epistasis (decelerating costs), presumably because they possess few non-pleiotropic biological functions, whereas large genomes tend to display negative epistasis (accelerating costs), possibly as a result of redundancy or mutational robustness [11]. However, whether or not costs associated with resistance and virulence alleles would interact in the same way as costs associated with random deleterious mutations is as yet largely unknown (although see ref. 13) and would be a fruitful avenue for future research.

Our findings have interesting implications for the Red-Queen Hypothesis (RQH) of the evolution of sexual reproduction in plants [12]. Typically GFG-type coevolution has not been considered compatible with the RQH, because it does not result in fluctuations of genotype frequencies. Sasaki [5] showed that such fluctuations were possible in a multilocus GFG model where costs of virulence and resistance were low. The findings presented here extend the range of conditions under which genotype frequency fluctuations are expected, and suggest that such fluctuations are more likely when epistasis is acceleratingly costly. Future empirical studies should attempt to quantify the interactive effects on fitness of multiple resistance and virulence genes. While such experiments are likely to be extremely difficult in natural populations, lab-based microbial host-parasite systems may provide important insights [13], [14], [15].

## Supporting Information

Figure S1 (4.19 MB TIF)

Figure S2 (4.19 MB TIF)

Figure S3 (4.19 MB TIF)

Figure S4 (4.19 MB TIF)

Figure S5 (4.19 MB TIF)

Figure S6 (4.19 MB TIF)

Supplementary Methods S1 (0.05 MB DOC)
