# Supplementary figures and images for: Epistatic Interactions Alter Dynamics of Multilocus Gene-for-Gene Coevolution
Source: PLoS One. 2007 Nov 7;2(11):e1156. doi: 10.1371/journal.pone.0001156 (PMC2065793; doi:10.1371/journal.pone.0001156)

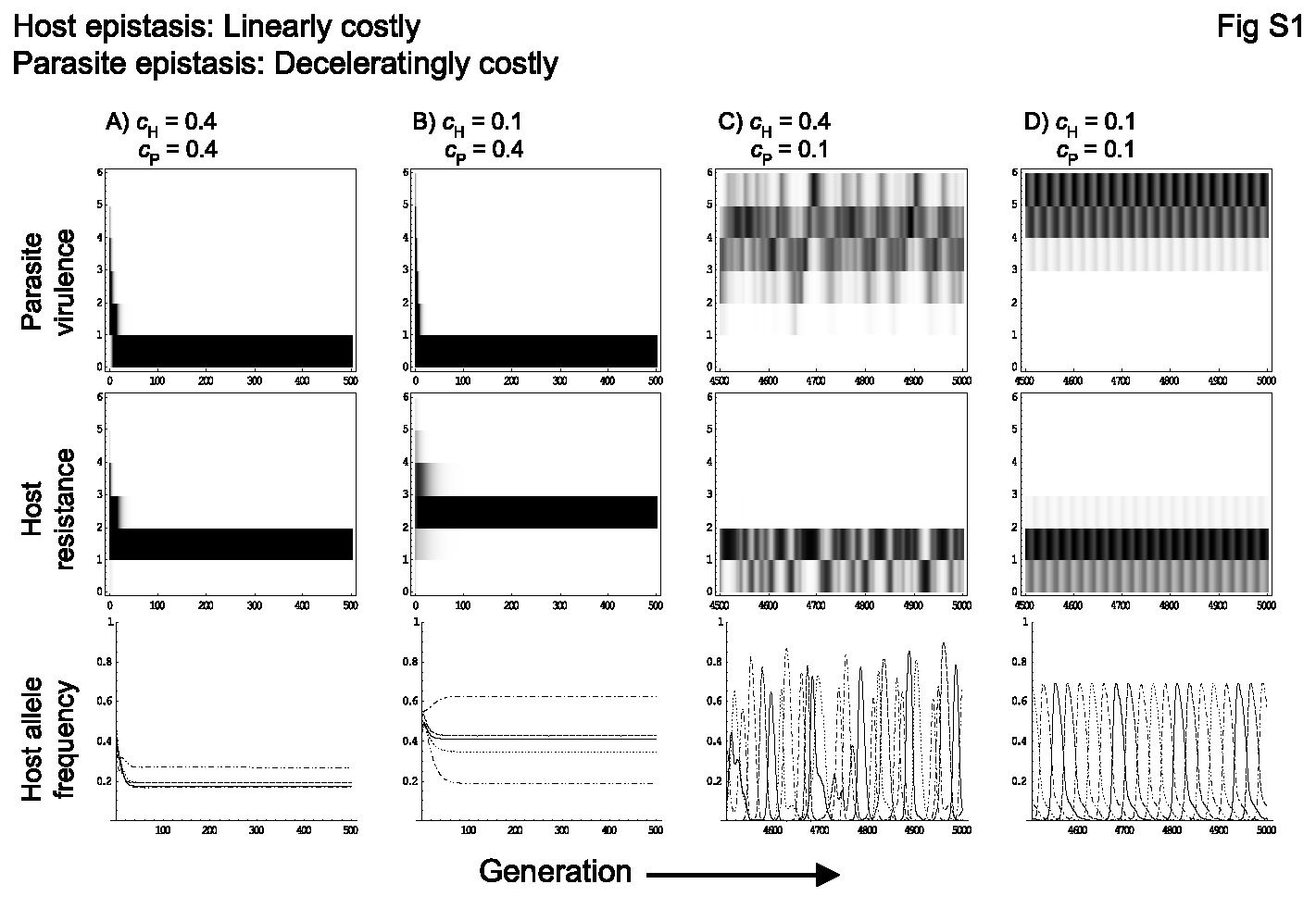

Supplement: Figure S1 — (4.19 MB TIF) [file pone.0001156.s001.tif]

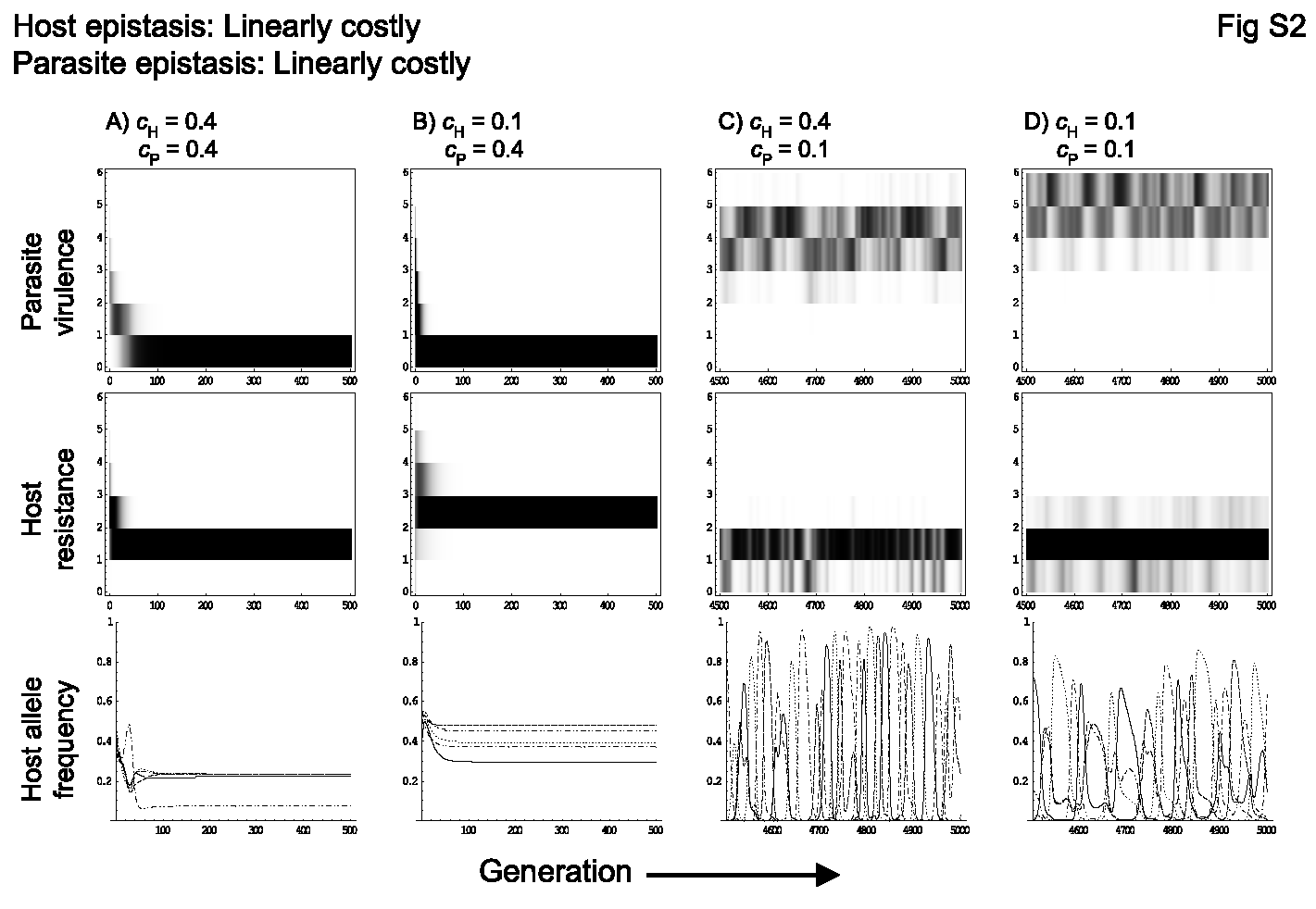

Supplement: Figure S2 — (4.19 MB TIF) [file pone.0001156.s002.tif]

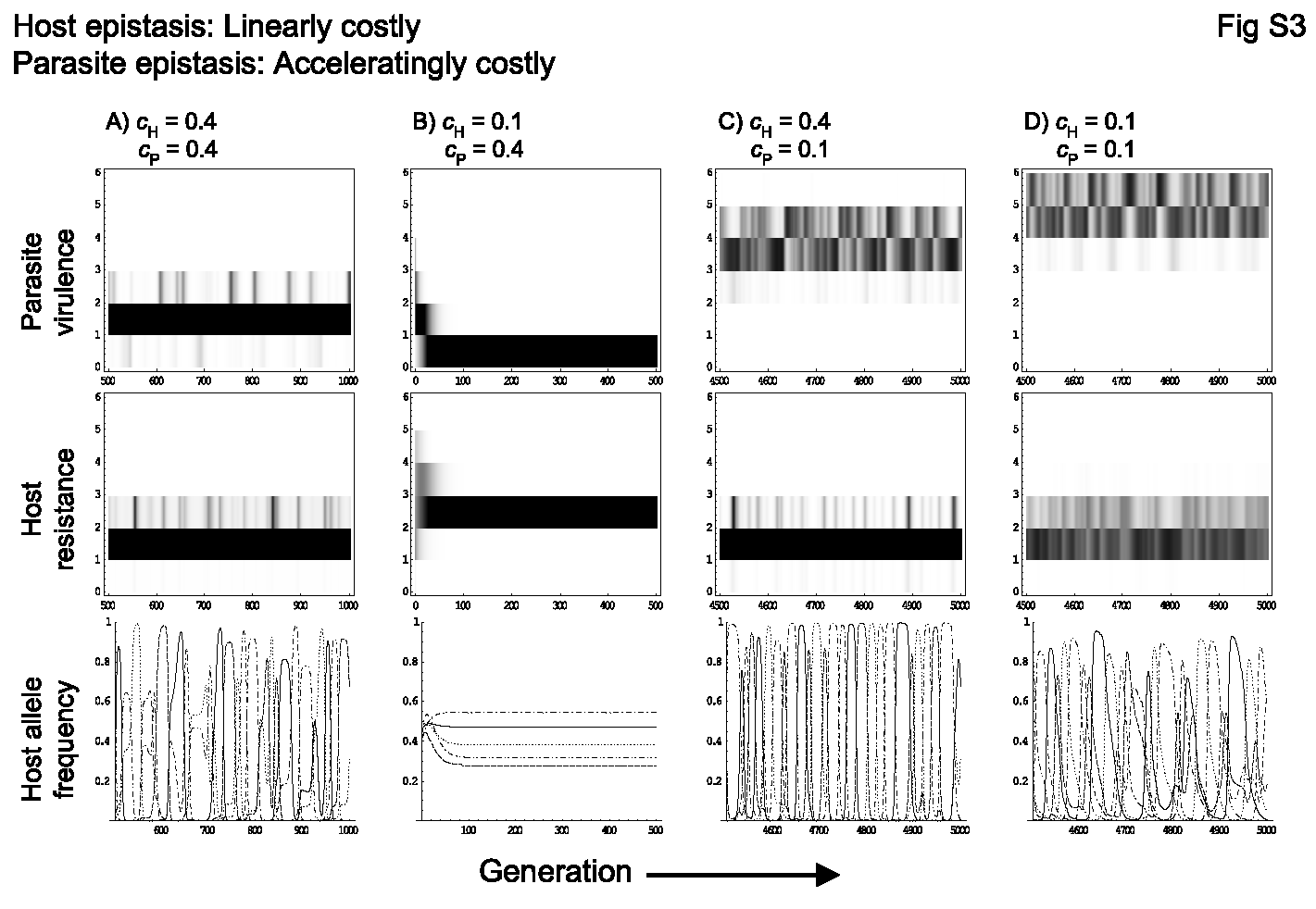

Supplement: Figure S3 — (4.19 MB TIF) [file pone.0001156.s003.tif]

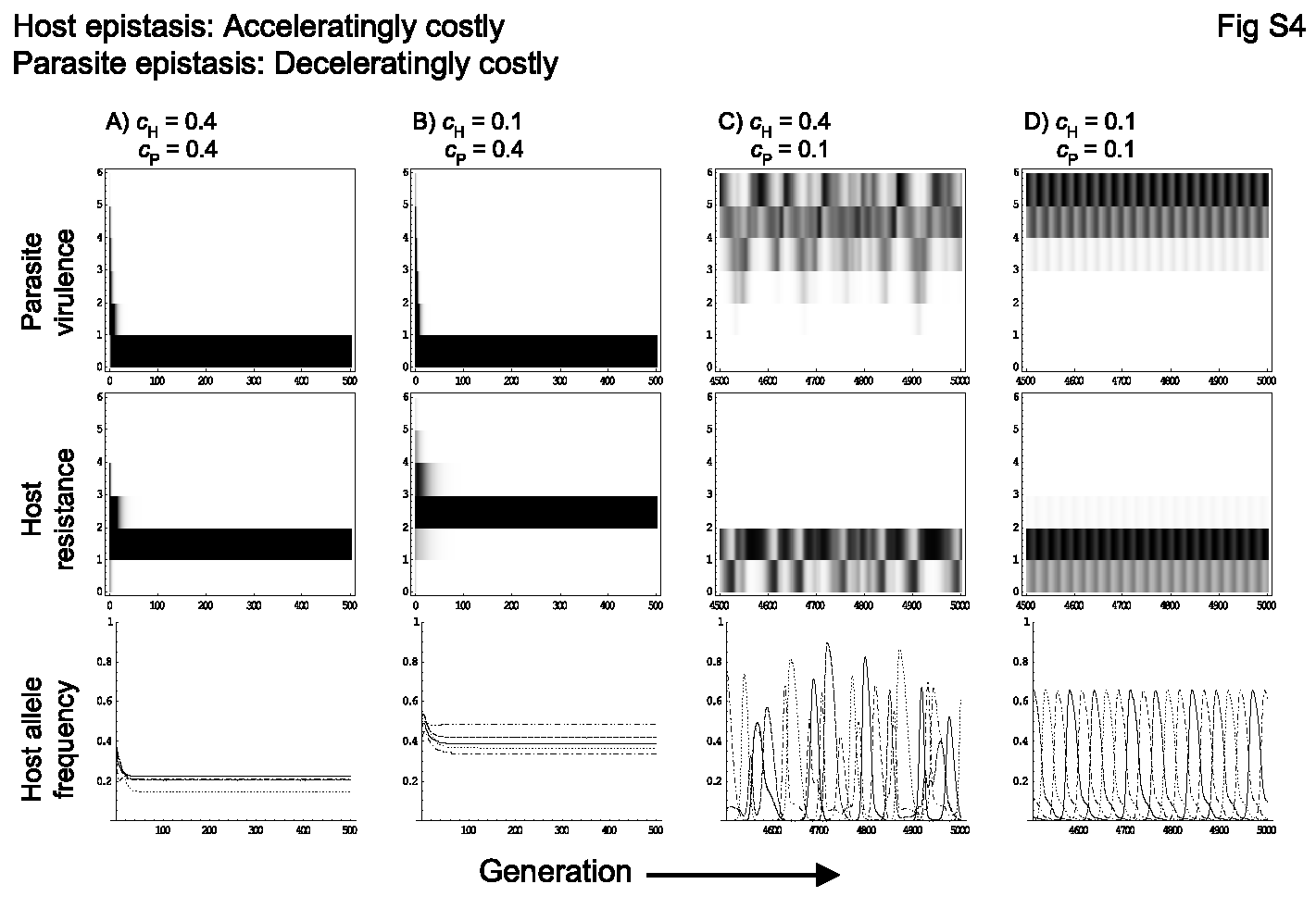

Supplement: Figure S4 — (4.19 MB TIF) [file pone.0001156.s004.tif]

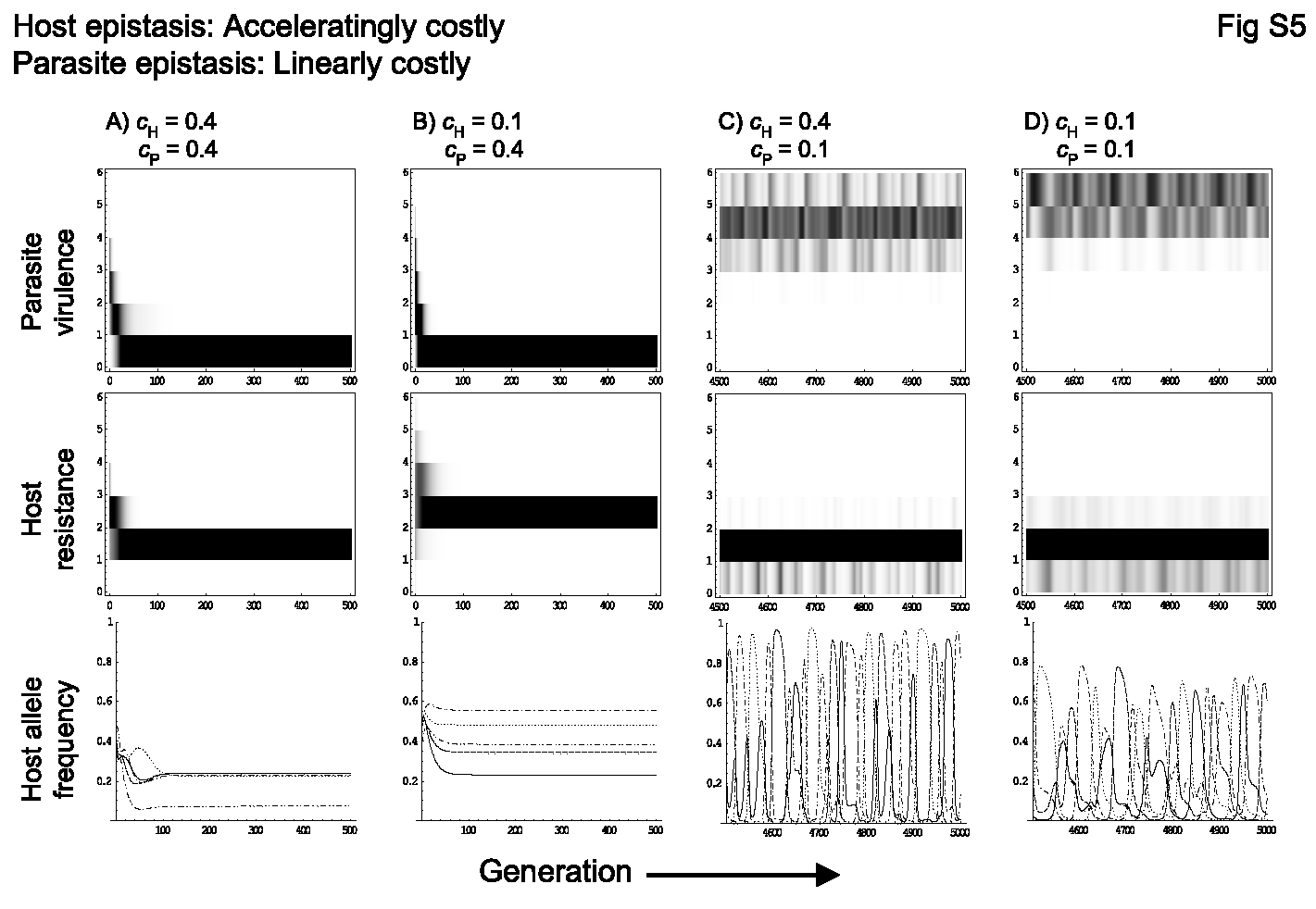

Supplement: Figure S5 — (4.19 MB TIF) [file pone.0001156.s005.tif]

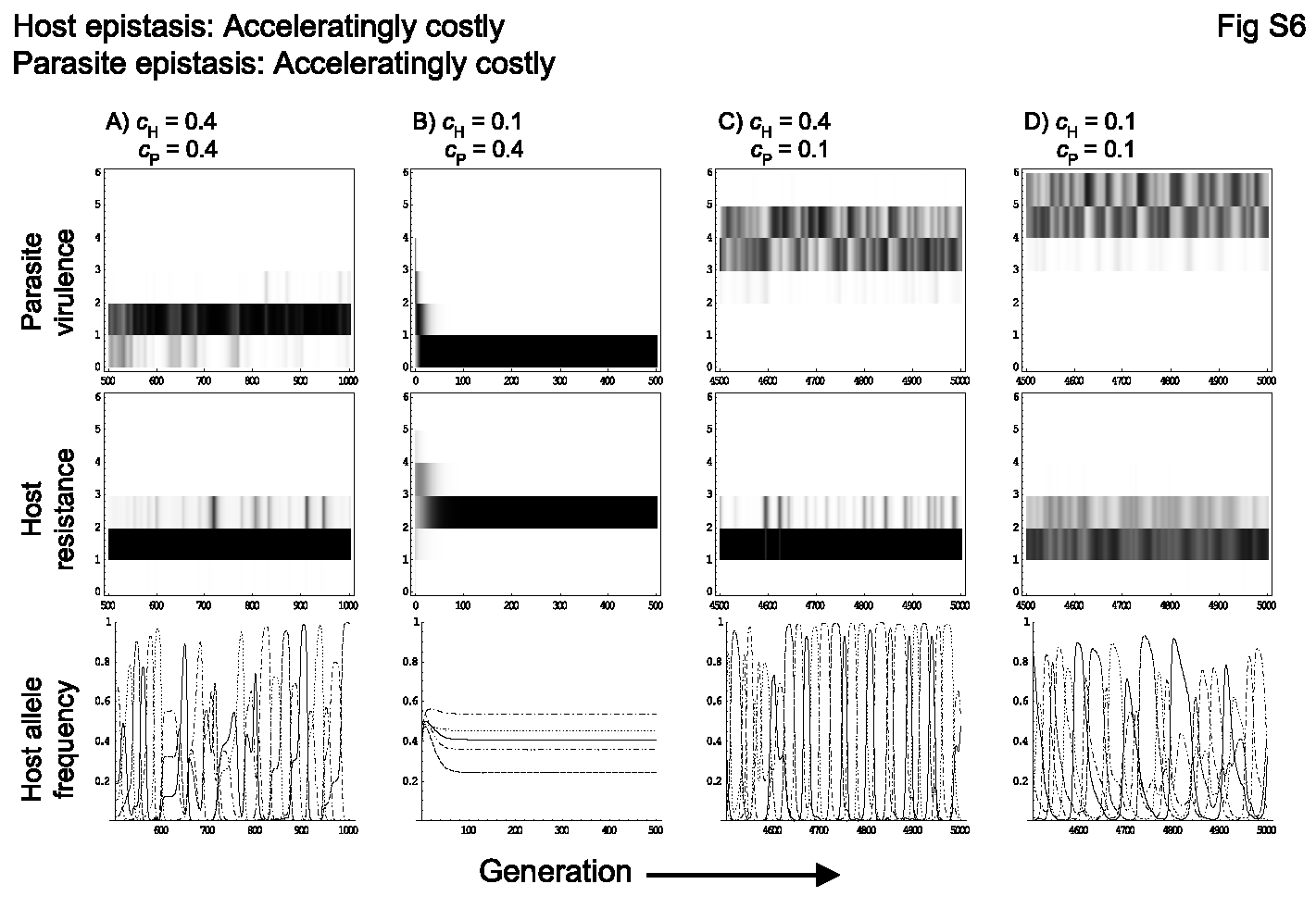

Supplement: Figure S6 — (4.19 MB TIF) [file pone.0001156.s006.tif]
