# Supplementary material for: Epistatic Interactions Alter Dynamics of Multilocus Gene-for-Gene Coevolution
Source: PLoS One. 2007 Nov 7;2(11):e1156. doi: 10.1371/journal.pone.0001156 (PMC2065793; doi:10.1371/journal.pone.0001156)
Supplement: Supplementary Methods S1 — (0.05 MB DOC) [file pone.0001156.s007.doc]

**ONLINE SUPPLEMENTARY MATERIAL**

**Epistatic interactions alter dynamics of multilocus gene-for-gene coevolution**

Andy Fenton & Michael A. Brockhurst

School of Biological Sciences, University of Liverpool, Crown Street, Liverpool, L69 7ZB.

**Model structure**

Our model is based on the multilocus gene-for-gene (GFG) model of Sasaki (2000). Here we outline the basic structure of the model common to both ours and that of Sasaki, leaving details of our modifications in the main paper. The model assumes there are *n* loci in the host which confer partial resistance to the parasite, and *n* corresponding virulence loci in the parasite which contribute towards successful infection. Each locus has one of two alleles; Resistant or Susceptible in the host and Virulent or Avirulent in the parasite. Hence, the host genotype for resistance is denoted by a string of binary numbers *s* = *s*_1_, *s*_2_, … , *s*_n_ and the parasite genotype by *t* = *t*_1_, *t*_2_, … , *t*_n_ where each digit is either a 1 or a 0, denoting Resistance or Susceptibility in the host or Virulence or Avirulence in the parasite respectively. The outcome of contact between and given host and a given parasite is determined by the combination of alleles at their respective resistance and virulence loci; partial resistance occurs if there is at least one combination of Resistant/Avirulent alleles at a single locus. In other words, partial resistance occurs if for at least one *i*, *s_i_* = 1 and *t_i_* = 0. Following Sasaki (2000), we use the notation *s* ≤ *t* to indicate genotype combinations where for all *i* any locus where *s_i_* = 1 is neutralised by a virulence allele (*t_i_* = 1).

The mean parasite load (ie. the probability of a given host individual with genotype *s* being infected following a random encounter with a parasite) is then $\sum_{t\geq s} p(t)$ where *p(t)* is the frequency of parasites with genotype *t* in the population. In other words, the probability of host genotype *s* being infected is simply the sum of parasite frequencies that are capable of infecting that host. The model then assumes that the fitness of host *s* decreases based on both its number of resistance genes due to a cost per gene, *c*_H_ of maintaining resistance and also due to the cost of being infected, at a per parasite rate *β*_H_. To incorporate partial resistance it is assumed that each effective resistance gene (ie. where *s_i_* = 1 and *t_i_* = 0) reduces the probability of successful infection to *σ*. If *r(s,t)* is the number of effective resistance genes of host genotype *s* when attacked by parasite genotype *t* then the probability of successful infection is

$Q(s,t) = \sigma^{r(s,t)}$.

The mean parasite load for host genotype *s* and the mean host availability for parasite genotype *t* are then $\sum_{t} \sigma^{r(s,t)}p(t)$ and $\sum_{s} \sigma^{r(s,t)}q(s)$ respectively. Hence, the fitness of host genotype *s* is:

$$w_{H}(s) = \exp\left\{-|s|c_{H}-\beta_{H}\sum_{t} \sigma^{r(s,t)}p(t)\right\}$$

where |*s*| is the number of resistance genes harboured by the host. Similar arguments show the fitness of parasite genotype *t* is:

$$w_{P}(t) = \exp\left\{-|t|c_{P}+\beta_{P}\sum_{s} \sigma^{r(s,t)}q(s)\right\}$$

where |*t*| is the number of virulence genes harboured by the host, *c*_P_ is the cost per virulence of gene, *β*_P_ is the fitness gain to the parasite of successful infection and *q*(*s*) is the frequency of hosts with genotype *s* in the population; the notation *s* ≤ *t* indicates host genotypes that parasite genotype *t* is able to infect.

Genotype frequencies change between generations due to selection according to:

$$q(s)' = w_{H}(s)q(s)/\bar{w}_{H}$$

and

$$p(t)' = w_{P}(t)p(t)/\bar{w}_{P}$$

where $\bar{w}_{H} = \sum_{s} w_{H}(s)q(s)$ and $\bar{w}_{P} = \sum_{t} w_{P}(t)p(t)$ are the mean fitnesses of hosts and parasites respectively in the population. In addition, there are assumed to be rare mutations, at rate *μ* between alleles at each locus each generation.

**Additional combinations of host and parasite epistasis curves**

In the main text we present results where the host species has deceleratingly costly epistasis associated with having increased genes for resistance and the parasite species has either deceleratingly costly, linear or acceleratingly costly epistasis associated with having increased genes for virulence. Here we present graphs showing the remaining combinations of hosts and parasites with deceleratingly, linearly or acceleratingly costly epistasis curves.

Fig S1. Coevolutionary host and parasite trajectories for different resistance (*c*B_HB_) and virulence (*c*B_PB_) costs, assuming linear epistasis for the host and deceleratingly costly epistasis for the parasite. The upper and middle panels show the frequency distributions over time of the number of virulence alleles in the parasite population and the number of resistance alleles in the host population, respectively. The bottom panels show the change in frequency of the host resistance alleles at each locus, where different line styles represent different loci. In all cases σ = 0.2, βB_HB_ = 1, βB_PB_ = 1 and μ, the mutation rate at each locus, was 2x10P^-5P^ per generation.

Fig S2. As for Figure S1, assuming linear epistasis for both the host and the parasite.

Fig S3. As for Figure S1, assuming a linear trade-off curve for the host and an acceleratingly trade-off curve for the parasite.

Fig S4. As for Figure S1, assuming acceleratingly costly epistasis for the host and deceleratingly costly epistasis for the parasite.

Fig S5. As for Figure S1, assuming acceleratingly costly epistasis for the host and linear epistasis for the parasite.

Fig S6. As for Figure S1, assuming acceleratingly costly epistasis for both the host and the parasite.
